# SVD-aided non-orthogonal decomposition (SANOD) method to exploit prior knowledge of spectral components in the analysis of time-resolved data

**DOI:** 10.1063/1.5085864

**Published:** 2019-03-26

**Authors:** H. Ki, Y. Lee, E. H. Choi, S. Lee, H. Ihee

**Affiliations:** 1 Center for Nanomaterials and Chemical Reactions, Institute for Basic Science (IBS), Daejeon 34141, South Korea; 2 Department of Chemistry and KI for the BioCentury, Korea Advanced Institute of Science and Technology (KAIST), Daejeon 305-701, South Korea

## Abstract

Analysis of time-resolved data typically involves discriminating noise against the signal and extracting time-independent components and their time-dependent contributions. Singular value decomposition (SVD) serves this purpose well, but the extracted time-independent components are not necessarily the physically meaningful spectra directly representing the actual dynamic or kinetic processes but rather a mathematically orthogonal set necessary for constituting the physically meaningful spectra. Converting the orthogonal components into physically meaningful spectra requires subsequent posterior analyses such as linear combination fitting (LCF) and global fitting (GF), which takes advantage of prior knowledge about the data but requires that all components are known or satisfactory components are guessed. Since in general not all components are known, they have to be guessed and tested via trial and error. In this work, we introduce a method, which is termed SVD-aided Non-Orthogonal Decomposition (SANOD), to circumvent trial and error. The key concept of SANOD is to combine the orthogonal components from SVD with the known prior knowledge to fill in the gap of the unknown signal components and to use them for LCF. We demonstrate the usefulness of SANOD via applications to a variety of cases.

## I. INTRODUCTION

Typical experimental data consist of signal elements that have different physical origins. For example, time-resolved spectra from transient absorption (TA) spectroscopy contain spectral contributions from various processes, such as absorption of reactants, absorption of excited states, and emission from excited states.^1–3^ In the case of time-resolved X-ray solution scattering (TRXSS), also known as time-resolved X-ray liquidography (TRXL),^4–29^ the data contain scattering contributions from the time-dependent structural changes of the solute molecules during a reaction and the time-dependent hydrodynamic response of the solvent. Moreover, experimental data are usually contaminated with random and systematic noise that emerges from the fluctuation of the experimental conditions.

Analysis of time-resolved data typically involves extraction of the time-independent components and determination of their time dependence by distinguishing between signal and noise. Singular value decomposition (SVD)^30–32^ is well suited for this purpose. However, the extracted time-independent components are not necessarily physically meaningful signals that directly represent actual dynamic or kinetic processes, but rather a mathematically orthogonal set necessary for constituting the physically meaningful spectra. SVD extracts (i) left singular vectors (LSVs), which are time-independent components that are required to reconstruct the observed data, (ii) right singular vectors (RSVs), which represent the time dependence of the LSVs, and (iii) singular values, which reflect the relative magnitudes of the contributions of the LSVs or RSVs to the data. However, the LSVs do not necessarily represent the absorption or emission spectra of certain states (for example, in the case of TA) or the scattering curves of chemical species (for example, in the case of TRXL) but are mathematically orthogonal components required for reconstructing the species-associated spectra or scattering curves (in the following discussion, the term spectra will be used to indicate both optical spectra and scattering curves). By contrast, the physically meaningful signal components, namely, the physical spectral components, are not necessarily orthogonal but are represented as linear combinations of the LSVs rather than in one-to-one correspondence to the LSVs.

For this reason, posterior analyses, such as principal component analysis (PCA), are often followed to convert the information from SVD into the spectra corresponding to the chemical species responsible for the observed data. In this stage, a model for the reaction kinetics has to be used as an input; thus, various models need to be tested if the reaction kinetics is unknown. Although numerous kinetics models may exist, the number can be reduced significantly through various methods, such as SVD-aided analysis using variable time ranges (V method) and SVD-aided pseudo-PCA (SAPPA).^33^

These methods work well for TRXL data on proteins because the hydrodynamic response signal from the solvent, which is also called the solvent heating signal, can be easily separated from the signal from proteins thanks to their different q regimes. As the solvent heating signal is significant only in a much larger q region than that for protein scattering signals, it can be removed from the original data to yield heat-free data. By contrast, in TRXL data on small molecules, the heating signal shares the same q range with the solute-related signal, except for some solvents with no hydrogen bonds, such as cyclohexane. Consequently, SVD or PCA is less effective in facilitating the data analysis.

We have observed that the solvent heating signals are quite reproducible and can be measured by a separate experiment using control molecules, such as a dye that does not accompany detectable molecular structural changes but only serves as a conduit to convey the photon energy to the solvent as heat.^34,35^ Naturally, such solvent heating signals can be exploited to facilitate the data analysis. A similar situation also occurs in other cases. For example, TRXL data collected at X-ray free-electron laser (XFEL) beamlines suffer from systematic noise due to the instability of the XFEL beams. Nevertheless, the systematic noise components can be obtained by SVD of the data at negative time delays and can thus be used as reliable prior knowledge.^36,37^ Similarly, if it is already known that a certain chemical process is involved, the scattering curves for the known process can be used as reliable prior knowledge. Methods that can fully exploit such prior knowledge would be highly beneficial compared to completely blind approaches that do not implement prior knowledge, such as SVD or PCA. Therefore, two additional methods are often used. In linear-combination fitting (LCF), the data at all the time points are individually processed independent of data at other time points and is decomposed into plausible components such as the scattering curves of candidate species and solvent heating signals. The relative contributions of all components obtained by LCF are later plotted as a function of the time delay to extract the kinetic information. Since the components are generally not known, they have to be guessed and tested via trial and error. In global fitting (GF),^7–10,16,20,22,36,38,39^ a candidate kinetic model and the time-dependent hydrodynamic response of solvent are used to fit data at all available time delays simultaneously. The advantage of GF compared to LCF is that the ratio of data points to fitting parameters increases considerably. As both the kinetics and the chemical species are unknown, various kinetic models have to be tested. For this reason, LCF is often applied to gain clues on the kinetic model and narrow down the candidate kinetic models to be tested for GF.

When one performs analysis of experimental data, it is rare to come across the case where a complete picture of all the signal components required to describe the experimental data is available. In other words, experimental data usually contain some signal components from unknown physical sources. In this case, only a part rather than a complete set of physical spectral components to describe an experimental data can be prepared. For LCF, the remaining unknown components need to be guessed and tested via trial and error. In this work, we introduce a method, which is termed SVD-aided Non-Orthogonal Decomposition (SANOD), to circumvent such a situation. The key concept of SANOD is to combine the orthogonal components from SVD with the known prior knowledge and to use them for LCF. In other words, the gap of the unknown signal components is filled by the orthogonal SVD components. SANOD can be applied in various cases to facilitate the analysis of experimental data. As a representative example, we show that SANOD can serve an effective noise and background filter to recover artifact-free experimental data when applied in a post-experimental treatment of experimental data contaminated by systematic artifacts. In addition, SANOD allows us to exploit the prior knowledge on the shapes of signal components such as solvent heating signals in TRXL data to facilitate the data analysis.

## II. THEORY OF SANOD

**FIG. 1.**
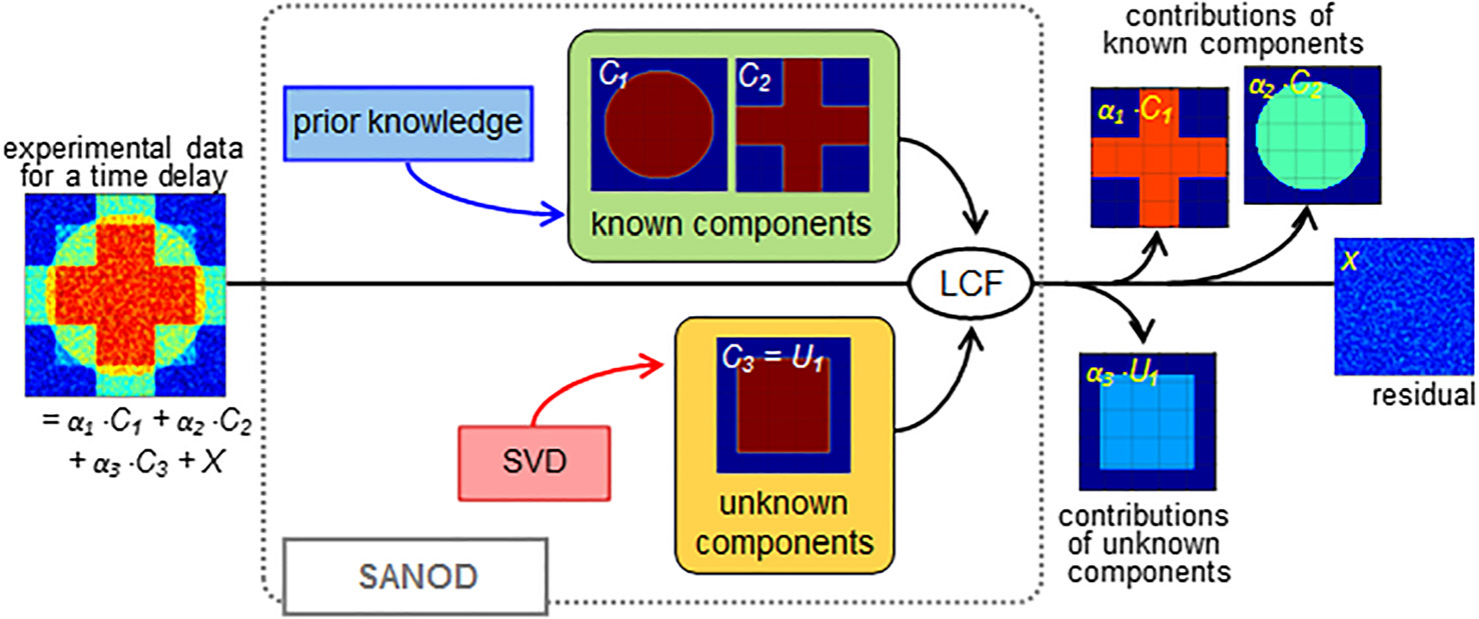
A schematic illustration of SANOD. The SANOD method is based on LCF of experimental data as the sum of the signal components. To prepare the signal components for SANOD, some of the components, i.e., the components that are already known to contribute to the data, are prepared by exploiting prior knowledge, while the other components to cover the contributions from unknown origins are supplied by SVD. By applying SANOD on the data, the unknown signal contributions can be separated from the contributions of the signal components having known physical origins and the residual signal. As a result, analysis of the unknown signal contribution is highly facilitated.

The concept of SANOD is illustrated in Fig. 1. The non-orthogonal decomposition of SANOD is based on LCF of experimental data as the sum of the signal components. To prepare the signal components for SANOD, some of the components are prepared by exploiting prior knowledge of the signal components, while the other unknown components are supplied by SVD. Here, we first outline the steps involved in LCF using the direct method, and a more detailed description is given in the supplementary material. The direct method refers to linear algebraic methods to yield the solution of equations without numerical iterative minimization.^40^

The data can be approximated as the sum of their time-independent spectral components as follows:

$$\begin{array}{c} \Delta S(q,\;t_{1})=\Delta S_{\mathrm{sum}}(q,\;t_{1})+X(q,\;t_{1})\\ =\alpha_{1}(t_{1})\cdot C_{1}(q)+\alpha_{2}(t_{1})\cdot C_{2}(q)+\cdots +\alpha_{n}(t_{1})\cdot C_{n}(q)+X(q,\;t_{1}),\\ \Delta S(q,\;t_{2})=\Delta S_{\mathrm{sum}}(q,\;t_{2})+X(q,\;t_{2})\\ =\alpha_{1}(t_{2})\cdot C_{1}(q)+\alpha_{2}(t_{2})\cdot C_{2}(q)+\cdots +\alpha_{n}(t_{2})\cdot C_{n}(q)+X(q,\;t_{2}),\\ \vdots \\ \Delta S(q,\;t_{n})=\Delta S_{\text{sum}}(q,\;t_{n})+X(q,\;t_{n})\\ =\alpha_{1}(t_{n})\cdot C_{1}(q)+\alpha_{2}(t_{n})\cdot C_{2}(q)+\cdots +\alpha_{n}(t_{n})\cdot C_{n}(q)+X(q,\;t_{n}), \end{array}$$ (1)

where $C_{i}(q)$ are the spectral components of the signal, $\alpha_{i}(t_{j})$ are their weights at time delay $t_{j}$, $\Delta S_{\text{sum}}(q,\;t_{j})$ is the approximated signal as the sum of the time-independent spectral components, and $X(q,\;t_{j})$ is a residual signal in $\Delta S(q,\;t_{j})$ for the approximation. When the spectral components, ${\left\{C_{i}(q)\right\}}_{i}$, are orthogonal as in the case of singular spectral modes in SVD analysis, the weight of a spectral component, $C_{i}(q)$, can be easily calculated using vector projection (see supplementary material for details). However, if ${\left\{C_{i}(q)\right\}}_{i}$ are not orthogonal, such a simple vector projection does not yield their exact weights in the data.

LCF using the direct method decomposes the data in terms of the initial input components, which are not necessarily orthogonal, and their weights. It is known that there are several direct method routines such as Gaussian elimination^40–45^ or QR decomposition^40,46,47^ to obtain the weights of the signal components. The former is known to be faster than the latter at the expense of accuracy and stability. Since accuracy and stability are more important than speed for our purpose, we decided to use QR decomposition as the means to decompose the experimentally observed signal into a sum of the input components, ${\left\{C_{i}(q)\right\}}_{i}$.

The LCF using QR decomposition involves calculating a set of orthonormalized signal components, ${\left\{O_{i}(q)\right\}}_{i}$, and using ${\left\{O_{i}(q)\right\}}_{i}$ to calculate the set of the weights, ${\left\{\alpha_{i}(t_{j})\right\}}_{i}$, of ${\left\{C_{i}(q)\right\}}_{i}$ at a certain time delay, $t_{j}$. Again, by repeating the procedure for the entire time series of experimental data, the time-dependent profiles of the contribution of each component, ${\left\{{\left\{\alpha_{i}(t_{j})\right\}}_{i}\right\}}_{j}$, are obtained. These profiles, which are called chronograms, show when and which processes occur during the reaction progress. The entire procedure to retrieve ${\left\{{\left\{\alpha_{i}(t_{j})\right\}}_{i}\right\}}_{j}$ is fully arithmetic, and therefore, the weights are calculated directly rather than obtained as a result of an iterative, maximum likelihood estimation procedure. Accordingly, the procedure is simple and fast and yields a unique optimal solution. With this said, it is worthwhile to note that the chronogram, ${\left\{{\left\{\alpha_{i}(t_{j})\right\}}_{i}\right\}}_{j}$, obtained from the direct method is the solution that minimizes the norm of each $X(q,\;t_{j})$ in Eq. (2). In other words, the method finds ${\left\{{\left\{\alpha_{i}(t_{j})\right\}}_{i}\right\}}_{j}$ that minimizes the magnitude of the residual signals; thus, the solution obtained from the direct method is the same as that obtained from least-squares minimization.^38^ Moreover, it is also possible to modify the direct method procedure in order to obtain ${\left\{{\left\{\alpha_{i}(t_{j})\right\}}_{i}\right\}}_{j}$ that minimizes the weighted least square, such as the chi-square, which exploits the experimental standard deviations. The modification and related discussions are presented in the supplementary material. In the demonstrations that follow, we use the modified direct method, which minimizes the chi-square of the residual signal. We note that the calculation time for the modified direct method increases compared to that for the original direct method because in the former case, the QR decomposition has to be repeated for the curves at all time delays whereas in the latter case, it needs to be performed for the curve at a single time point and the orthogonalized components can be reused for the curves at the other time points.

As mentioned earlier, experimental data normally contain some signal components from unknown physical sources. Accordingly, only a part rather than a complete set of physical spectral components to describe experimental data can be prepared based on prior knowledge. To fill in the missing signal components, SANOD borrows the spectral components obtained from SVD. The SANOD method is as follows. First, let the physical spectral components which can be prepared from known physical origins be ${\left\{K_{i}(q)\right\}}_{i}$. Second, by applying SVD to the entire experimental data, one can identify the number and shape of significant singular spectra. Let the significant singular spectra obtained from SVD be ${\left\{U_{j}(q)\right\}}_{j}$. The key idea of SANOD is to use the combined set of ${\left\{K_{i}(q)\right\}}_{i}$ and ${\left\{U_{j}(q)\right\}}_{j}$ for decomposing experimental data. More specifically, if we assume the number of components in ${\left\{K_{i}(q)\right\}}_{i}$ to be *n* and the number of components in ${\left\{U_{j}(q)\right\}}_{j}$ to be *m*, then ${\left\{C_{i}(q)\right\}}_{i}$ in Eq. (2) is composed as follows:

$${\left\{C_{i}(q)\right\}}_{i=1\;\text{to}\;n}={\left\{K_{i}(q)\right\}}_{i},$$ (2)

$${\left\{C_{n+j}(q)\right\}}_{j=1\;\text{to}\;m}={\left\{U_{j}(q)\right\}}_{j}.$$ (3)

After the preparation of ${\left\{C_{i}(q)\right\}}_{i}$, LCF using the direct method is applied to the experimental data using ${\left\{C_{i}(q)\right\}}_{i}$. Before employing ${\left\{C_{i}(q)\right\}}_{i}$ directly for SANOD, it is necessary to ensure whether the components in ${\left\{C_{i}(q)\right\}}_{i}$ are linearly independent. Specifically, it should be confirmed that there is no component that can be expressed as a linear combination of the other components in ${\left\{C_{i}(q)\right\}}_{i}$. If such a component exist, it should be discarded from ${\left\{C_{i}(q)\right\}}_{i}$ because it causes Eq. (1) to have non-unique but infinitely many solutions because its contribution is not unique but can be replaced by the contributions of the other components. Applying QR decomposition to ${\left\{C_{i}(q)\right\}}_{i}$ containing such linearly dependent components yields some meaningless $O_{i}(q)$, which are zero vectors or only have noise fluctuations and meaningless ${\left\{{\left\{\alpha_{i}(t_{j})\right\}}_{i}\right\}}_{j}$ .

At first glance, the procedure of SANOD seems to be complicated because it requires both SVD and LCF to be performed for the analysis. Nevertheless, this method has a considerable advantage compared to relying only on SVD. Through SANOD, the signal constituents having known physical origins, i.e., the contribution of ${\left\{K_{i}(q)\right\}}_{i}$ components, can be separated from the signal contributions from unknown origins. This approach thus facilitates analysis and identification of the unknown origins that affect the experimental signal. The details of the applications will be presented in the demonstrations with two different types of real experimental data that contain signal components with unknown origins, but before, we present these examples of SANOD, we first demonstrate the advantage of LCF over SVD in terms of extracting correct kinetics and signal components and the importance of correct input components for a successful LCF.

## III. RESULTS AND DISCUSSION

### A. Demonstration of the advantage of LCF using the prior knowledge over SVD

**FIG. 2.**
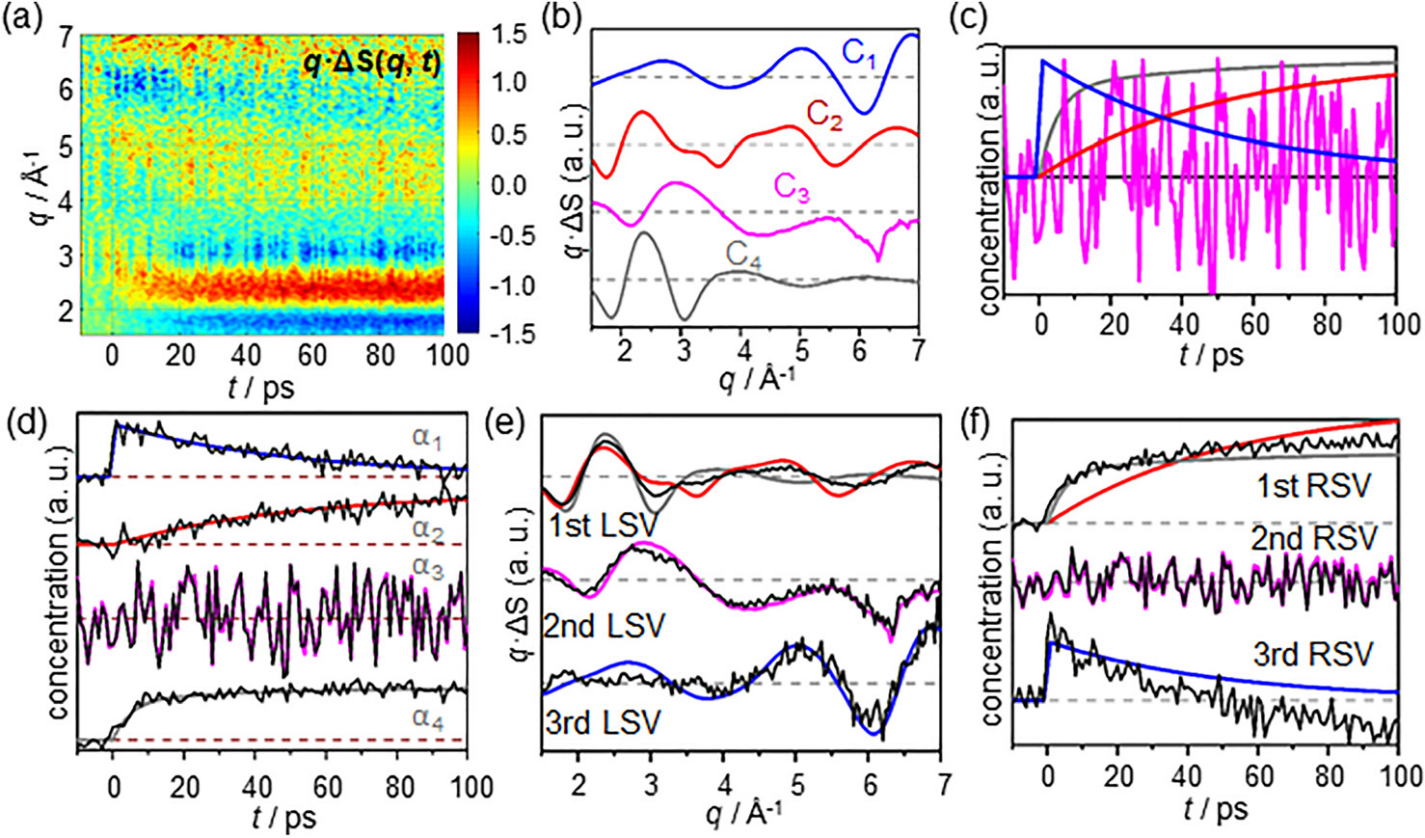
Demonstration of SANOD using mock time-resolved data. (a) Mock TRXL data. (b) The mock data shown in (a) are prepared by using four different signal components, which represent the structural changes of solute to form an early intermediate (blue solid line) and a product (red solid line), a systematic, experimental artifact (magenta solid line), and hydrodynamic response of solvent (gray solid line). In addition to the four components, a random noise with about 6% of the magnitude of the other components is added to the mock data. (c) The time-dependent weights, or the chronograms, for the four components [shown in (b)] used to generate the mock data. (d) The comparison of the chronograms retrieved from SANOD (black solid lines) and their original chronograms (colored lines) which are the same as those shown in (c). The agreement is excellent. (e) The three significant LSVs (black solid lines) obtained by applying SVD to the mock experimental data. For comparison, the original four signal components (colored lined) which are the same as those shown in (b) are also shown. The agreement is not so good except the 2nd LSV, of which shape is similar to the reference for the systematic artifact probably because this component is present throughout the time range. (f) The three RSVs (black solid lines) for the three significant LSVs are overlapped with the original chronograms. The agreement is not so good except the 2nd RSV.

To demonstrate the advantage of LCF using the prior knowledge over SVD in terms of extracting physically meaningful spectra, we prepared mock TRXL data of a hypothetical chemical reaction. A two-step sequential reaction that involves an instantaneous formation of an intermediate species and a subsequent transformation into a product was assumed. Based on the reaction scheme, the mock data of the reaction consist of four different signal components [see Figs. 2(a) and 2(b)]. Each component has a different physical origin; one is from the heating of a solvent, another is from an experimental systematic artifact, and the other two are from the structural changes of the solute molecules to form the intermediate species and the product. Among these four signal components, the latter two contain information on the structural changes during the reaction and therefore are of interest, whereas the former two are not in general. The reference chronograms of the four components used to generate the mock data are shown in Fig. 2(c). In addition to the four components, random noise with around 6% intensity of the signal was added to the mock data.

Under the assumption that the shapes of all the signal components are known, LCF was applied to the mock data to obtain the chronograms of the components. The result shown in Fig. 2(d) confirms that the chronograms calculated using LCF are in excellent agreement with the original chronograms used to generate the mock data. This may seem obvious, but such separation of the mixture of signal components is difficult to achieve using a method such as SVD, which does not implement prior knowledge on the experimental data. To demonstrate this point, SVD was also applied to the mock data for comparison with the LCF results. With SVD, only three major singular spectra are obtained, rather than the four components used to create the mock data. Among the three singular spectra, the shapes of two singular spectra, the 1st and 3rd LSVs, differ considerably from the shapes of any of the reference signal components, whereas the shape of the other singular spectrum is similar to that of the reference for the systematic artifact [see 2nd LSV in Fig. 2(e)] probably because this component is present throughout the time range. In fact, the two RSVs corresponding to the 1st and 3rd LSVs also show considerable differences from the reference chronograms [see Fig. 2(f)]. Such differences are expected because a singular component obtained from SVD does not represent a physical behavior but a mathematically orthogonal element that can constitute experimental data.

### B. Demonstration of the importance of correct signal components for a successful LCF

As mentioned previously, when preparing the signal components for SANOD, some of the components are prepared by using prior knowledge of the signal components, while the other unknown components are supplied by SVD. The reason for this complicated process is that LCF does not properly estimate the weight of each signal component unless the set of signal components, ${\left\{C_{i}(q)\right\}}_{i}$, for the LCF is correctly configured so that the experimental data can be satisfactorily explained as the linear combination of the components. Otherwise, if ${\left\{C_{i}(q)\right\}}_{i}$ is not properly prepared, i.e., a component to explain the experimental data is missing in ${\left\{C_{i}(q)\right\}}_{i}$, LCF gives some unreliable weights of the components.

**FIG. 3.**
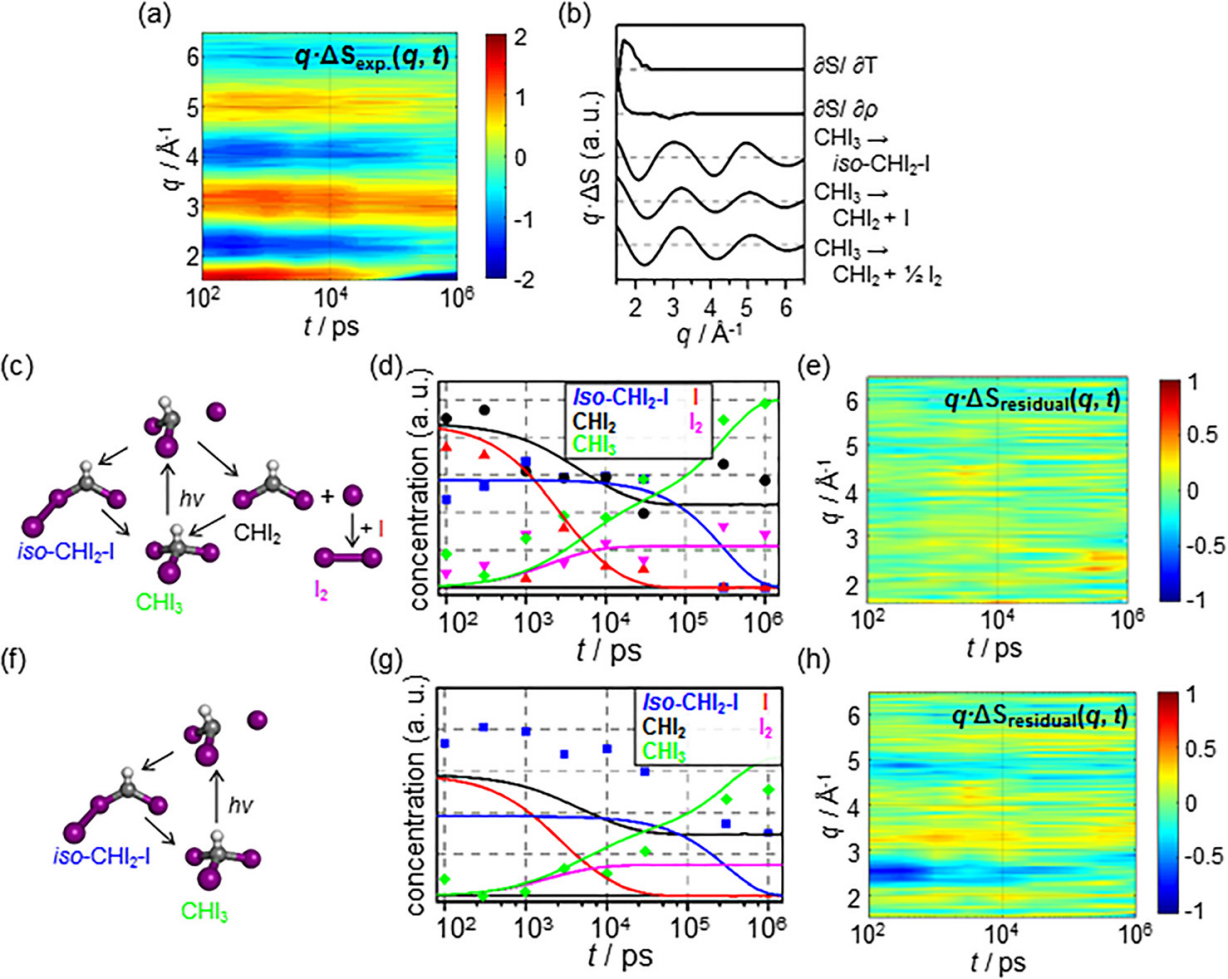
An example to show the importance of correct signal components for LCF. (a) The experimental TRXL data obtained from the photoreaction of CHI_3_ molecules in cyclohexane. (b) The five signal components corresponding to the reaction model. (c) The photoreaction pathways of CHI_3_ which were identified by analyzing the TRXL data. The first two components represent the hydrodynamic response of cyclohexane solvent [(∂S(*q*)/∂T)_*ρ*_ and (∂S(*q*)/∂*ρ*)_T_], and the other three represents the scattering curves due to the relevant species involved in the reaction. (d) The chronograms (scatter plots) of the reaction intermediates obtained by LCF based on the reaction model shown in (c). The chronograms obtained using LCF agree well with the concentration profiles reported from the previous study (solid lines). (e) The residual signal of LCF analysis. The residual is negligible when compared to the raw experimental data shown in (a). (f) Another candidate photoreaction pathway of CHI_3_ which lacks the long-lived CHI_2_ species. (g) The chronograms (scatter plots) of the reaction intermediates obtained via LCF based on the reaction model shown in (f). The chronograms obtained via LCF no longer agree with the concentration profile reported from the previous study (solid lines). (h) The residual signal of LCF analysis which is based on the reaction model shown in (f). There are considerable magnitudes of residuals when compared to (e). Note that the color scale in (e) and (h) is reduced by a factor of 2 when compared to (a) in order to improve the visibility of the residual signal.

To demonstrate this point, LCF was applied to TRXL data obtained from a synchrotron for the photoreaction of CHI_3_ in cyclohexane [see Fig. 3(a)]. Previously, there was ambiguity on the reaction pathway, especially on the kinetics of the formation and the decay of the long-lived radical intermediate, CHI_2_, in the course of reaction. Recently, the reaction pathways, which include the long-lived radical intermediate, were identified by analyzing the TRXL data through an iterative trial-and-error based GF analysis of different candidate models.^22^ According to the best-fitting model for the reaction pathways revealed from the study, shown in Fig. 3(c), the experimental data are represented as the sum of five different components; two are from the hydrodynamic response of the solvent [(∂S(*q*)/∂T)_*ρ*_ and (∂S(*q*)/∂*ρ*)_T_], and the other three are from the structural changes of the molecules during the reaction [see Fig. 3(b)].

When LCF is applied using the five components (a proper ${\left\{C_{i}(q)\right\}}_{i}$) following the established kinetic model, the experimental data can be successfully decomposed to yield only negligible amounts of residuals [see Fig. 3(e)]. It can be seen that the chronograms obtained using LCF are in good agreement with the concentration profile of each species, which were reported in the previous study. A modified kinetic model omitting the pathways involving the long-lived radical species can be compared using LCF [see Fig. 3(f)]. For this kinetic model, the experimental data are expressed as the sum of three different components because there is only a single component that corresponds to the structural change of the molecules. When LCF is applied using these three components which lack two signal components, the chronograms [Fig. 3(g), scatter plots] no longer agree with the concentration profiles reported in the previous study. In addition, the residuals of the LCF are no longer negligible but have considerable magnitudes [see Fig. 3(h)].

It is often debated as to which kinetics model most satisfactorily describes a reaction among a number of plausible candidate models. In such cases, one of the best ways to settle the debate is to test the candidate models against the experimental data and identify the most suitable one. Toward this end, LCF can be employed to expedite the comparison of the candidate kinetic models that fit the experimental data. As shown in Eq. (1) and Fig. 3, experimental data are decomposed as a sum of the components, $\Delta S_{\text{sum}}(q,\;t)$, and a residual, X(q, t). The better the shapes and the number of signal components according to a candidate model for fitting the experimental data are the closer the $\Delta S_{\text{sum}}(q,\;t)$ value is to ΔS(q, t) and the smaller is the residual. To verify a model on the basis of these criteria, X(q, t) of the model can be calculated rapidly and accurately with the aid of LCF using the direct method. This demonstration also underlines that the use of LCF greatly eases the comparison by facilitating the retrieval of the residual corresponding to a model.

### C. Practical applications of SANOD: Experimental data with unknown signal components

#### 1. Noise filtering of experimental data contaminated by systematic artifacts

A representative application of SANOD is post-experimental treatment of experimental data to filter out systematic artifacts having reproducible spectral shapes. Ideally, it is desirable to establish a perfect experimental condition that excludes all the sources of artifacts in the data. However, the establishment of such an ideal situation is often hindered by technical or theoretical limitations. In some experiments, where the experimental conditions are not stable but fluctuating, the magnitude of such artifacts can be sufficiently large to be comparable with or to overwhelm the desirable signal from the sample. As a representative example, the state-of-the-art femtosecond TRXL experiments at XFEL facilities typically suffer from considerable systematic noise that contaminates the desirable signal from the sample. In such cases, an appropriate post-experimental treatment to remove these artifacts is critical for the success of data analysis.

If the shapes of the artifacts are known, they can be used as ${\left\{K_{i}(q)\right\}}_{i}$. Through SANOD using these ${\left\{K_{i}(q)\right\}}_{i}$, the contribution of the artifacts can be separated and removed from the experimental data. Accordingly, the first step in the post-experimental treatment of the data contaminated with the artifacts is to retrieve the shape of the artifacts. A representative method for this purpose is to use data at negative time delays.^36,37^ It was reported that the components obtained by using SVD of the data at negative time delays can be used for the corrections of the artifacts in the data via LCF.^36^ At negative time delays, the reaction is not yet initiated; hence, there should be no signal contribution related to the progress of the reaction. Therefore, any features detected in the negative time delays can be regarded as artifacts originating from an imperfect experimental setup. In the previously reported method,^36^ however, since the contribution of unknown signal components had to be guessed for this purpose, the possibility that the resultant signal, where the known artifacts are removed, could be distorted cannot be ruled out.

**FIG. 4.**
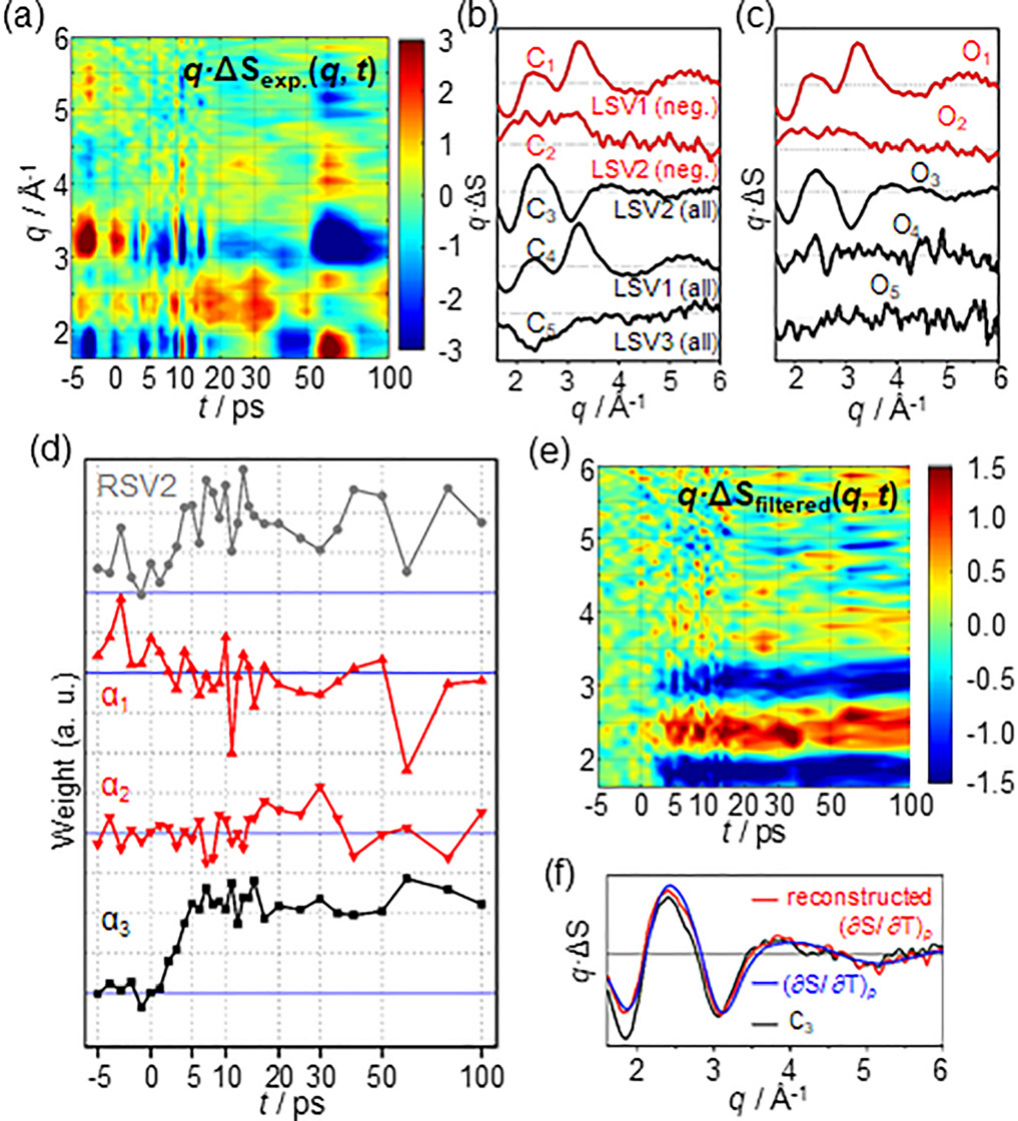
A demonstrative example showing how experimental data contaminated by systematic artifacts can be treated with SANOD. (a) Real TRXL data measured at an XFEL beamline used for the demonstration. The data are for a dye molecule dissolved in water and are corrupted by experimental artifacts which originate from some fluctuations of experimental conditions. There are considerable, noisy fluctuations in the experimental data even in the negative time delays. (b) The spectral components prepared for SANOD analysis of the experimental data. The upper two components ($C_{1}$ and $C_{2}$, red solid lines) represent the experimental artifacts which are the first and second LSVs obtained by using SVD of a part of experimental data that correspond to negative time delays, *t *<* *0 ps. The lower three components ($C_{3}$, $C_{4}$, and $C_{5}$, black solid lines) are the second (LSV2), the first (LSV1), and the third (LSV3) significant LSVs obtained by SVD of the whole experimental data. (c) The result of QR decomposition of $C_{i}$. After orthonormalization, only meaningless noises remain in $O_{4}$ and $O_{5}$, which means that $C_{4}$ and $C_{5}$ can be expressed as linear combinations of the other components. Therefore, for the subsequent SANOD analysis, only $C_{1}$, $C_{2}$, and $C_{3}$ are used. (d) The chronograms obtained by SANOD. The results confirm that $\alpha_{1}(t)$ and $\alpha_{2}(t)$, the chronograms for the systematic artifacts, show noisy fluctuations around zero weight. In contrast, the chronogram for $C_{3}$, $\alpha_{3}(t)$, which should represent the solvent heating response, shows a clear rise after the photoexcitation. $\alpha_{3}(t)$ obtained by using SANOD is in contrast to that from SVD alone (gray), which is shown in the uppermost panel for comparison. (e) The experimental data after filtering out the contribution of the artifacts, $C_{1}$ and $C_{2}$. The filtered data were prepared by subtracting $\alpha_{1}(t)\;\cdot \;C_{1}$ and $\alpha_{2}(t)\;\cdot \;C_{2}$ from the raw data. The rise of difference scattering intensities after photoexcitation is now clearly visible. Note that the color scale is reduced by a factor of 2 when compared to (a) in order to improve the visibility of the residual signal. (f) Comparison of the spectral shape of the signal from hydrodynamic response of water [(∂S(*q*)/∂T)_*ρ*_, blue] to that of $C_{3}$ (black). The shapes of the two are similar but different. ∂S(*q*)/∂T)_*ρ*_ is expressed rather as a linear combination of $C_{1}$, $C_{2}$, and $C_{3}$ (red) than as $C_{3}$ alone and can be reconstructed by a simple PCA using the constraint that the contributions of $C_{1}$ and $C_{2}$ should fluctuate around zero as described in Sec. C of the supplementary material.

To verify the post-experimental data treatment using SANOD, the method was applied to TRXL experimental data corrupted by experimental artifacts. The experiment was performed at an XFEL facility, SPring-8 Angstrom Compact Free-Electron Laser (SACLA), to examine the kinetics of the hydrodynamic response of water as a solvent. The data considered were for a dye molecule (4-amino-1,1′-azobenzene-3,4′-disulfonic acid monosodium salt) dissolved in water. Upon laser excitation, the excited dye molecules dissipate their energies to the surrounding water molecules, leading to an increase in the temperature of the solvent. The data measured from the experiment are shown in Fig. 4(a). Large and noisy fluctuations can be observed over the entire time delays along with clear oscillatory artifacts even in the negative time delays. As a result, the change in the difference scattering signal owing to the increase in solvent temperature is hardly visible.

To obtain the shapes of the artifacts, the difference scattering curves for *t* < –2 ps were analyzed using SVD. The result showed that there are two major spectral components that comprise the artifact at negative time delays [see Fig. 4(b)]. The two spectral components are used as ${\left\{K_{i}(q)\right\}}_{i}$, which represent the spectral contribution of noisy artifacts. To compose ${\left\{U_{i}(q)\right\}}_{i}$ in order to describe the other signal contributions, SVD was applied to the entire set of experimental data spanning from negative to positive time delays. Three significant spectral components were obtained from SVD [see Fig. 4(b)] and were used as ${\left\{U_{j}(q)\right\}}_{j}$. Note that as the set ${\left\{U_{j}(q)\right\}}_{j}$ consists of the significant components for the entire experimental data, it alone is sufficient to describe both the artifacts and the other signal contributions from the sample. However, in each of the three components in ${\left\{U_{j}(q)\right\}}_{j}$, the contributions of the noisy artifacts and the signal from the sample are not separated but mixed together owing to the nature of SVD. Now, the set ${\left\{C_{i}(q)\right\}}_{i}$ for SANOD consists of five components; two components describe the experimental artifacts, and the other three describe the signal from the sample.

Before employing ${\left\{C_{i}(q)\right\}}_{i}$ directly for SANOD, it is necessary to ensure whether the components in ${\left\{C_{i}(q)\right\}}_{i}$ are linearly independent. ${\left\{C_{i}(q)\right\}}_{i}\;$ just prepared for the noisy TRXL data in this demonstration can be an excellent example. When ${\left\{C_{i}(q)\right\}}_{i}$ is orthogonalized using the QR decomposition, two components, $O_{4}(q)$ and $O_{5}(q)$, become meaningless noise, as shown in Fig. 4(c), and all ${\left\{{\left\{\alpha_{i}(t_{j})\right\}}_{i}\right\}}_{j}$ are corrupted (not shown). It means that each of the two components, $C_{4}(q)$ and $C_{5}(q)$, can be expressed as a linear combination of $C_{1}(q)$, $C_{2}(q)$, and $C_{3}(q)$. Therefore, these two components are discarded from ${\left\{C_{i}(q)\right\}}_{i},$ and only the three components $C_{1}(q)$, $C_{2}(q)$, and $C_{3}(q)$ are used for SANOD.

The resulting chronograms of the three components shown in Fig. 4(d) confirm that the smoothly rising chronogram of the desired signal from the sample, $\alpha_{3}(t)$, is retrieved from the noisy experimental data. The chronogram apparently well describes the kinetics of heat transfer from the dye to the solvent. The temperature of the solvent indeed remains constant prior to the laser excitation and increases upon the laser excitation. Note that the smooth profile is in clear contrast to the noisy RSV of the component and thus demonstrates the power of SANOD as a method for filtering out experimental artifacts. The chronograms of the experimental artifacts $\alpha_{1}(t)$ and $\alpha_{2}(t)$ show noisy fluctuations around zero.

On the basis of the two chronograms, the contribution of the artifacts can be removed from the experimental data by subtracting $\alpha_{1}(t)\cdot \;C_{1}(q)$ and $\alpha_{2}(t)\cdot {\;C}_{2}(q)$. The resulting filtered experimental data are shown in Fig. 4(e). The filtered experimental data are clean at negative time delays, which confirms that the artifacts are successfully filtered out from the experimental data. It can also be seen that the intensity of the difference scattering curves increases gradually after time zero. The result definitely demonstrates the power of SANOD as a means of noise filtering. We note that in this process, a part of the unknown signal component (in this case, (∂S(*q*)/∂T)_*ρ*_) and all artifacts are removed. This is because the filtered data should be composed of $C_{3}(q)$, which is $U_{1}(q)$ and not identical to (∂S(*q*)/∂T)_*ρ*_. The reason $U_{1}(q)$ is not the same as (∂S(*q*)/∂T)_*ρ*_ is because $U_{1}(q)$ is from the SVD of the entire data. It should be noted that $\alpha_{3}(t)$ is precisely known via SANOD and thus can be used to retrieve the unknown signal component (in this case, (∂S(*q*)/∂T)_*ρ*_) as explained in Sec. C of the supplementary material, where we shows the proof that $\alpha_{3}(t)$ obtained for $U_{1}(q)$ is the same as the kinetic trace for (∂S(*q*)/∂T)_*ρ*_. Since $\alpha_{3}(t)$ is known, a simple PCA using the constraint that the contributions of $C_{1}(q)$ and $C_{2}(q)$ should fluctuate around zero can retrieve (∂S(*q*)/∂T)_*ρ*_.

We also tested the effect of errors in the prior knowledge by replacing the correct heating signal with the intentionally distorted heating signal containing errors and compared the results with those of the case using the correct heating signal. Figure S2 (supplementary material) shows $\alpha_{3}(t)$ traces for various cases. As the amount of error increases, $\alpha_{3}(t)$ deviates more from $\alpha_{3}(t)$ obtained from the correct (∂S(*q*)/∂T)_*ρ*_ or $U_{1}(q)$, and accordingly, the residual increases. Nevertheless, the correct (∂S(*q*)/∂T)_*ρ*_ can be still obtained using the residual as described in Fig. S2 and Sec. D of the supplementary material. As demonstrated earlier, the correct (∂S(*q*)/∂T)_*ρ*_ can be retrieved without the prior knowledge of the exact shape of (∂S(*q*)/∂T)_*ρ*_ by using $U_{1}(q)$. Therefore, these simulations illustrate that (i) the correct (∂S(*q*)/∂T)_*ρ*_ can be still obtained by using either $U_{1}(q)$ (as described in Sec. C of the supplementary material) or the residual (as described in Sec. D of the supplementary material) and (ii) it is more convenient to use $U_{1}(q)$ instead of the known (∂S(*q*)/∂T)_*ρ*_ to retrieve the correct (∂S(*q*)/∂T)_*ρ*_.

In a previous study,^48^ the intensity and the energy profiles were monitored for each shot and used to find correlations with the scattering data at negative time delays. The source of noise could be identified and used to remove artifacts from the data because the intensity and energy were additionally measured to find the correlations with the measured data. However, this method can remove only the noise additionally monitored. For example, if the liquid jet fluctuation is another source of noise, this method requires an additional simultaneous measurement to monitor the jet stability, i.e., the thickness and the position of the liquid jet, to remove the associated noise. The SANOD method is different in that it does not require such additional simultaneous measurements and can be used even when the sources of noise are not known.

#### 2. Kinetic analysis of an experimental signal

The purpose of this demonstration is to show how SANOD can be used to facilitate the kinetic analysis of experimental data. In general, experimental data are a combination of several signal components, each having different kinetics and thereby showing a complicated progression over time. When such experimental data contain some signal components with known spectral shapes, SANOD can be applied to facilitate the kinetics analysis. Specifically, by applying SANOD, the contributions of such components can be separated from the experimental data. Consequently, the kinetic analysis of the remaining data, which consist of a smaller number of components, is simplified considerably.

**FIG. 5.**
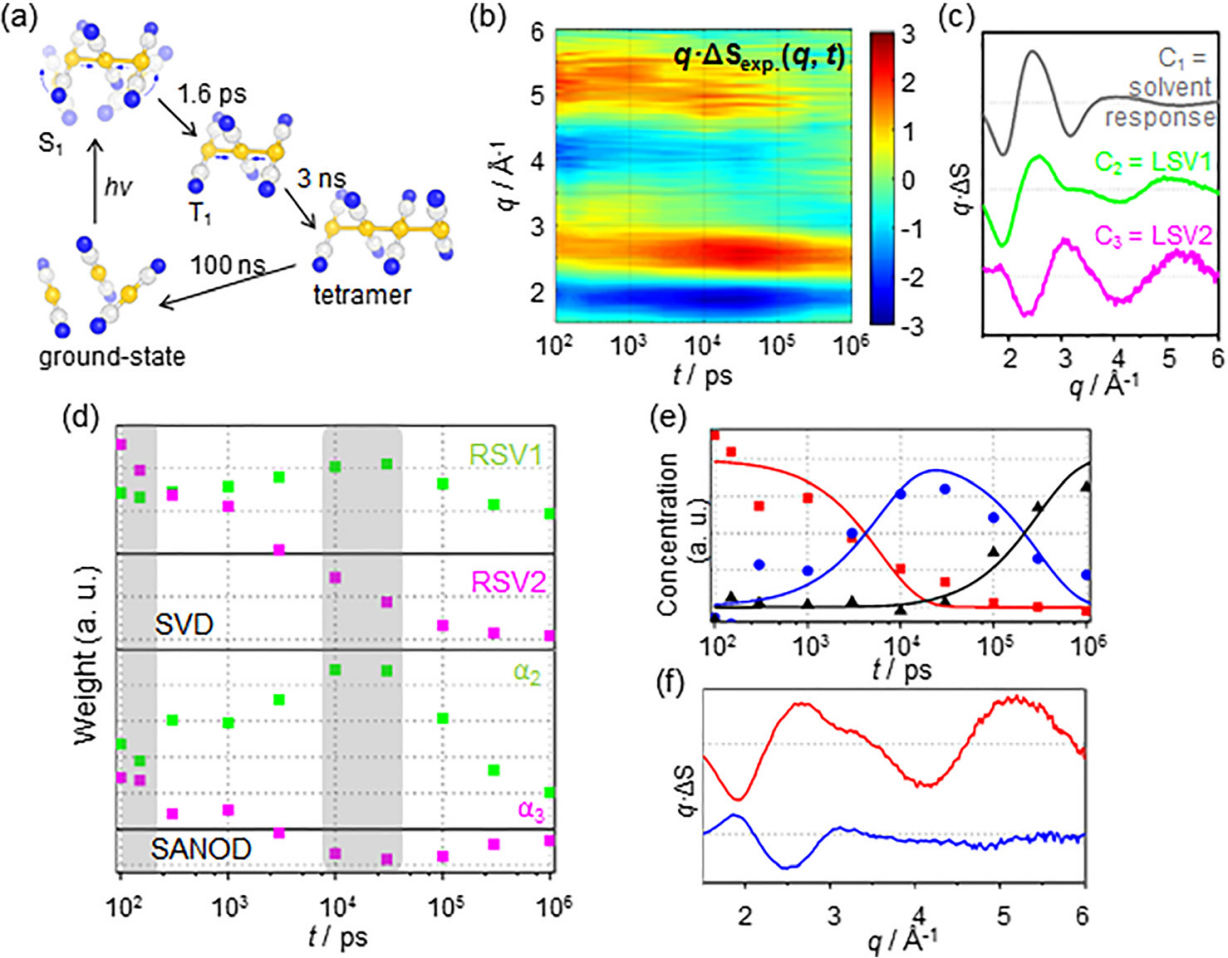
A demonstrative example to show how SANOD can be used to facilitate the kinetic analysis of experimental data. (a) A schematic diagram for the photoreaction pathways of Au(CN)_2_^−^ trimers in water which were revealed from the analysis of a TRXL study.^20,49^ (b) The TRXL experimental data for the photoreaction of Au(CN)_2_^−^ trimers in water. (c) The signal components used for SANOD analysis of the experimental data. The first component, $C_{1}$ (gray solid line), is for the solvent response of the water solvent. For SANOD analysis, the known (∂S(*q*)/∂T)_*ρ*_ of water was used for $C_{1}$. For $C_{2}$ (green solid line) and $C_{3}$ (magenta solid line), the two significant LSVs obtained by using SVD of the experimental data were used. (d) The chronograms obtained as a result of SANOD analysis (green squares for $\alpha_{2}$ and magenta square for $\alpha_{3}$, lower panel). There are two stationary time zones (gray colored regions), the regions where the amplitudes of all the chronograms remain constant, for the chronograms at around 100 ps and 10 ns. For comparison, the RSVs corresponding to the two components, $C_{2}$ and $C_{3}$, are plotted in the upper panel. It can be seen that there is no stationary time zone for the RSVs, in contrast to the chronograms obtained by using SANOD. (e) The species-associated chronograms (red squares for an early intermediate, blue circles for the second intermediate, and black triangles for the recovery of the ground state) obtained by using SAPPA which extracts the reaction kinetics with the aid of the detected stationary time zones from (d). The chronograms are in good correspondence with the kinetics of the reaction which was identified from the previous study (solid lines). (f) The extracted chronograms were then used to the subsequent PCA analysis. The resulting two species-associated spectral components are shown.

To demonstrate this aspect, SANOD was applied to data [see Fig. 5(b)] measured from a synchrotron for the photoreaction of Au(CN)_2_^−^ trimers in water. Even though the detailed structural dynamics regarding the photoreaction has already been published [see Fig. 5(a)],^20,49^ we assumed that the number of intermediate species (two intermediates, i.e., the trimer in the T_1_ state and the tetramer) and their structures identified in previous studies are not yet known. This was intended to mimic a typical situation at the beginning of the analysis of the experimental data.

As for the physical spectral components with known shapes, ${\left\{K_{i}(q)\right\}}_{i}$, the hydrodynamic response of water as a solvent was used. In addition, as for ${\left\{U_{j}(q)\right\}}_{j}$, the two significant singular spectra obtained from SVD were used [see Fig. 5(c)]. In contrast to the example in C-1, ${\left\{K_{i}(q)\right\}}_{i}$ and ${\left\{U_{j}(q)\right\}}_{j}$ are linearly independent (not shown). Consequently, the experimental data were decomposed by SANOD using a total of three spectral components. The lower panel of Fig. 5(d) shows two chronograms corresponding to the two ${\left\{U_{i}(q)\right\}}_{i}$ components, i.e., unknown contributions. In the chronograms, there are two stationary time zones,^33^ i.e., regions where the amplitudes of all the chronograms remain constant, at around 100 ps and 10 ns. When retrieving kinetic information from experimental data, such stationary time zones provide important clues for identifying kinetic models of a reaction.^33^ The chronograms obtained from SANOD are in clear contrast to those obtained from SVD, which do not have any stationary time zone. The hydrodynamic response of the solvent is responsible for the contrasting tendencies in the chronograms. While there is no structural change of the molecules, the shape of the difference scattering curves continues to change owing to the increase in the temperature of the solvent. Therefore, in the SVD result, in which the contributions of the hydrodynamic response of the solvent and the structural change of the molecules are not separated, the stationary time zone is not detected. By contrast, through SANOD, it is possible to filter out the known contributions of the solvent heating to yield the remaining signal containing information on “unknown molecular changes.” Based on the observed stationary time zones in the chronograms, a kinetic model of the reaction can be established. Using the kinetic model, species-associated physical spectral components [see Fig. 5(f)] and their chronograms [see Fig. 5(e)] can be retrieved, as is usually done in a pseudo-PCA analysis such as SAPPA.^33^ The results confirm that the obtained chronograms are in excellent correspondence with the kinetics of the reaction, which was identified from the previous study [see Fig. 5(e), solid lines]. The shapes of the physical spectral components are similar to those of the experimental data at around 100 ps and 10 ns but different because the contribution of the solvent is removed in the physical spectral components. The structural information corresponding to each species can be retrieved by analyzing the species-associated physical spectral components.

### D. Comparison of SANOD and GF

Comparison of calculation times between SANOD and GF is not straightforward due to the dependence on the number of time points. For the mock data shown in Fig. 2, which consist of 111 time delays and 145 spectral points and four spectral components, GF (0.141 s) takes about 6.4 times more than SANOD (0.022 s). The difference would become smaller as the number of time points increases, but SANOD would be faster than GF as far as the number of time points is not huge. We note that the calculation time for SANOD given here is for the modified SANOD of which the target solution is that of chi-squared minimization. If the original SANOD, of which the target solution is that of least-squares minimization, is used, the calculation time gets even shorter (0.0036 s) because for the original SANOD, the QR decomposition needs to be performed for the curve at a single time point, and the orthogonalized components can be reused for the curves at the other time points. We do not expect that SANOD can replace GF as they are complementary. A typical scenario is to apply SANOD to a dataset to remove systematic noise and gain insights into candidate kinetic models, which are then tested by GF. On the other hand, structural parameters are usually refined with GF, but SANOD can be also used for the same purpose if combined with an iterative fitting routine.

## IV. CONCLUSION

In this work, we introduce a method named SANOD which takes advantage of known prior knowledge for the analysis of time-resolved data and demonstrate that the SANOD can be applied to facilitate the analysis of TRXL data on small molecules. SANOD fills in the missing unknown information by using orthogonal components from SVD and combines them with known components from prior knowledge. As a result, the contributions of known signal origins such as experimental artifacts, hydrodynamic response of solvents, or known chemical processes can be easily separated out from the experimental data even if not all components are known. Therefore, SANOD can be used as an efficient and accurate aid to the conventional means to analyze the data. In this work, we showed various application examples for TRXL data, but, in principle, SANOD should be applicable to data from other types of experimental techniques.

## SUPPLEMENTARY MATERIAL

See supplementary material for discussions regarding (i) the detailed description of the formulas related to SANOD, (ii) the relationship between SANOD and least-squares minimization, (iii) the modified SANOD procedure for obtaining the chronograms that minimize the weighted least square, (iv) the method of posterior analysis to retrieve the correct spectral shape of the components after SANOD, (v) the proof that the chronogram is the same regardless of whether $U_{1}(q)$ or (∂S(*q*)/∂T)_*ρ*_ of water is used as the third component for SANOD in the example of the application of SANOD in Sec. III C 1, and (vi) the method to retrieve the correct shape of the signal component from erroneous prior knowledge. The TRXL data on CHI_3_ in cyclohexane were collected at the ID09B beamline in ESRF. The TRXL data on Au(CN)_2_^−^ trimers in water were collected at the NW14A beamline in KEK. The TRXL data experiments on the dye in water conducted as part of this study were performed at the BL3 beamline of SACLA. The experimental details are also provided.
